# Utility of fetal facial markers on a second trimester genetic sonogram in screening for Down syndrome in a high-risk Thai population

**DOI:** 10.1186/s12884-021-04332-0

**Published:** 2022-01-11

**Authors:** Savitree Pranpanus, Kanokkarn Keatkongkaew, Manaphat Suksai

**Affiliations:** Maternal and Fetal Medicine Unit, Department of Obstetrics and Gynecology, Faculty of Medicine, Prince of Songkla University, Hat Yai, Songkhla, 90110 Thailand

**Keywords:** Prenasal thickness, Nasal bone length, Prenasal thickness to nasal bone length ratio, Nasal bone to prenasal thickness ratio, Facial sonomarkers, Second trimester, Down syndrome screening

## Abstract

**Background:**

To establish the reference ranges and evaluate the efficacy of the fetal facial sonomarkers prenasal thickness (PT), nasal bone length (NBL), PT/NBL ratio and NBL/PT ratio for Down syndrome screening in the second trimester of high-risk pregnancies using two-dimensional (2D) ultrasound.

**Methods:**

A prospective study was done in Thai pregnant women at high risk for structural and chromosomal abnormalities between May 2018 and May 2019. The main exclusion criteria were any fetal anatomical anomaly detected on ultrasonography or postpartum examination, abnormal chromosome or syndrome other than Down syndrome. Ultrasounds were performed in 375 pregnant women at 14 to 22 weeks’ gestation and the fetal facial parameters were analyzed. Down syndrome results were confirmed by karyotyping. The reference ranges of these facial ultrasound markers were constructed based on the data of our population. The Down syndrome screening performance using these facial ultrasound markers was evaluated.

**Results:**

In total, 340 euploid fetuses and 11 fetuses with Down syndrome met the inclusion criteria. The PT, NBL, and PT/NBL ratios in the euploid fetuses gradually increased with gestation progression while the NBL/PT ratio gradually decreased between 14–22 weeks’ gestation. The NBL, PT/NBL ratio, and NBL/PT ratio all had 100% sensitivity and PT had 91% sensitivity. These facial markers had 100% negative predictive value for Down syndrome screening in the second trimester. The Bland–Altman analysis showed the intra- and inter-observer variations of PT and NBL had high intraclass correlation coefficients (ICC) in both operators, with ICCs of 0.98 and 0.99 and inter-observer ICCs of 0.99 for both operators.

**Conclusion:**

The facial ultrasound markers are very useful for second trimester Down syndrome screening in our population. These facial ultrasound markers were easily identifiable and highly consistent either intra- or inter-operator by using widely-available 2D ultrasound. However, the reference ranges for these markers need to be constructed based on individual populations.

**Trial registration:**

Registration number: REC 61–029-12–3. Date of registration: 18 May 2018.

## Background

Down syndrome, first described in 1866, is one of the most common fetal aneuploidies [1]. The typical Down syndrome profile includes a flat face with a flat nose and subcutaneous skin edema. Abnormalities of the lymphatic vessels result in variable degrees of skin edema and an increasing of skin thickness in particular areas such as the face and nuchal fold [2, 3].

These common collective findings can thus be used as a screening tool for fetuses with Down syndrome in the first [3–6] and second trimesters [7–11]. There have been reports of some facial ultrasound markers that may be useful in the second trimester for Down syndrome screening, for example the absence or hypoplasia of the nasal bone, prenasal thickness (PT), frontomaxillary facial angle and prefrontal space ratio (PFSR) [10, 12–19]. All of these facial ultrasound markers can be measured with one standard ultrasound midsagittal fetal facial view in the second trimester [20]. These measurements are not difficult to perform, and various studies have found the acquisition of these images and the resulting measurements to have good reproducibility [11, 16].

There is strong evidence that an absent or hypoplastic nasal bone is a potential marker for Down syndrome screening in the second trimester [9, 21, 22]. However, this marker has differences in the normal ranges among races and ethnicities [8, 11, 12], and when using reference ranges from other countries the detection rate has been found to be lower than expected in Asians [23]. Recently, the PT to NBL (PT/NBL) ratio has been widely reported as the most effective sonomarker for Down syndrome screening [7, 10, 11, 24–26]. These studies reported detection rates of PT/NBL for Down syndrome of 86–100%. However, there is only one study from Asia, which was done in Chinese women, which found a lower detection rate of 46% for PT/NBL for Down syndrome screening when using 3D ultrasound [15], although the mean PT/NBL ratio in euploid fetuses and cut-off for Down syndrome screening were different from previous studies [7, 12, 15, 24, 25].

In this study, our objective was to establish the reference ranges and evaluate the screening performance of PT, NBL, PT/NBL ratio and NBL/PT ratio for Down syndrome screening in second trimester women in our Thai population. We also wanted to examine the possible extra benefits of facial ultrasound markers in identifying women at high risk for aneuploidy and/or structural abnormalities.

## Methods

A prospective study was carried out in 375 singleton, high-risk-pregnancies who underwent ultrasound scans at 14–22 weeks’ gestation between May 2018 and May 2019. The criteria for high-risk pregnancy were advanced maternal age (≥ 35 years), presence of one or more soft ultrasound markers from a previous ultrasound, a previous history of aneuploidy or congenital abnormality fetuses and/or patients identified as high risk for having a Down syndrome fetus following a national quadruple screening test in Thailand. The study was approved by the Ethics Committee of the Faculty of Medicine, Prince of Songkla University, Thailand. All of the study women were provided information about the purposes and methods of the study and provided written informed consent before undergoing their ultrasounds.

The gestational age was calculated from a first or second trimester scan before 20 weeks gestation. Fetal karyotyping was performed if amniocentesis was indicated. In participants with no karyotyping, the neonatal outcomes were determined from post-delivery pediatrician reports. The exclusion criteria were non-singleton pregnancies, non-Thai ethnicity, any anatomical anomaly detected on ultrasonography or postpartum examination, abnormal chromosome or syndrome other than Down syndrome, miscarriage or fetal death before 24 weeks, and/or neonatal outcomes information not available.

### Measurements

**Fig. 1:**
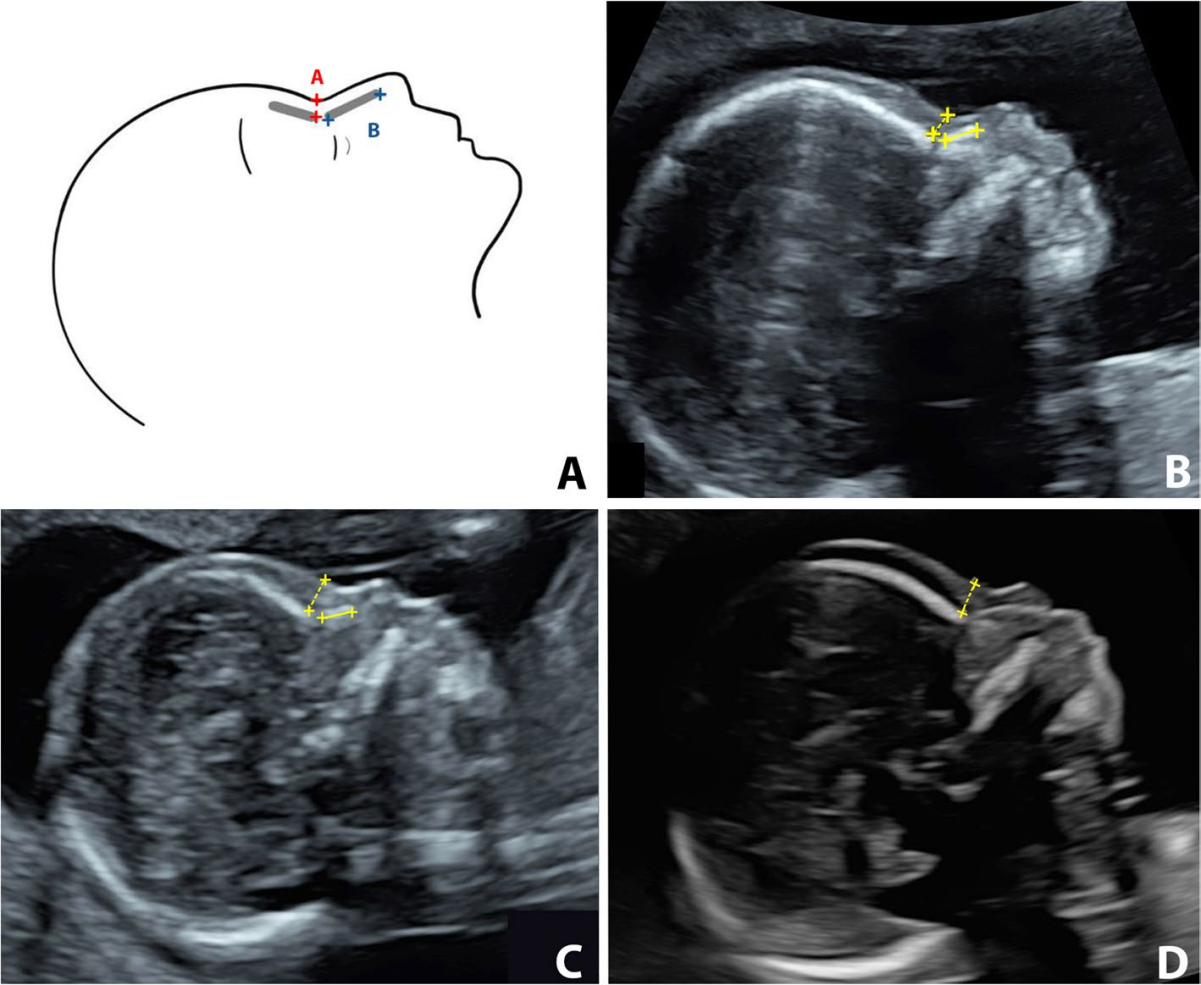
Measurement of mid-facial plane facial ultrasound markers. **Legend:** The landmarks used for measurement of the facial ultrasound markers in the study are shown in **(A)**. PT is the distance between the edge of the lowest part of the frontal bone and the facial skin anteriorly **(A)**, NBL is measured at the ossification line along the fetal nose **(B)**. Also shown are ultrasound images of the mid-facial plane in a euploid fetus **(B)** and a Down syndrome fetus with a hypoplastic nasal bone **(C)** and absent nasal bone **(D)**. PT: prenasal thickness; NBL: nasal bone length.

Each woman was scanned by 2-dimensional (2D) transabdominal ultrasound, with a GE Voluson S10 or Voluson E8 system equipped with a RAB4-8L probe (GE Medical Systems, Kretz Ultrasound, Zipf, Austria). The first 100 cases were done by two sonographers with different degrees of experience; one had experience of less than five years and the second one had experience of over 5 years. This was done with the intent to evaluate the agreement between the two sonographers. The rest of the participants were scanned by one of these two sonographers. Each sonographer took 10 min for scanning each participant and if the measurements could not be achieved in that time, that participant was excluded. Each pregnant woman was scanned only one time. The sonographers were unaware of the karyotype because all the participants underwent their ultrasound before amniocentesis. The measurements were performed according to the criteria described originally by Maymon et al. [12]. The exact mid-sagittal plane was defined according to the standard landmarks of the forehead, tip of the nose, visible translucent diencephalon or corpus callosum and palate without a zygomatic bone. PT was measured at the shortest distance between the anterior edge of the lowest part of the frontal bone and the facial skin anteriorly, as shown in Fig. 1. NBL was measured from the junction of the nasal and frontal bones to the distal edge of the white ossification line. These two parameters were measured 3 times in each fetus and the average of the 3 measurements was used for the final analysis for Down syndrome screening.

### Statistical analysis

Maternal demographic and ultrasonographic data and neonatal outcomes were recorded in Microsoft Excel. Statistical analyses were performed using Stata v14.2. Continuous variables were calculated as mean ± SD. The distributions of PT and NBL (in mm), and PT/NBL and NBL/PT ratios according to gestational age were visualized using scatter plots. Using the euploid fetuses data, linear regression models for each marker (PT, NBL, PT/NBL and ln[NBL/PT]) were constructed against gestational age represented by days and days^2^ to predict mean values according to gestational age. Predicted percentiles (5^th^ and 95^th^) for each marker were constructed from mean marker level ± 1.64*, the standard deviation of the residuals obtained from the corresponding linear regression model. The percentile values of PT, NBL and PT/NBL and exponentiated percentiles of ln(NBL/PT) together with the original data of the euploid fetuses were plotted against gestational age. Marker levels from the fetuses with Down syndrome were added to the graphs to compare with the levels from the euploid fetuses. Using the 5^th^ and 95^th^ percentiles according to the marker concerned, the predictive properties of each marker were estimated.

The agreements between the two measurements of each observer were evaluated from the first 100 cases during the study period. The PT and NBL were evaluated, and intraobserver and interobserver agreements calculated using Bland–Altman analysis and intraclass correlation coefficient (ICC). In all analyses, P < 0.05 was considered to be statistically significant.

## Results

A total of 375 pregnant women who underwent ultrasound in our high-risk clinic were enrolled in this study. A sagittal view of the fetal face was obtained within 10 min from a 2D ultrasound in 99.7% of the participants. Twenty-four women were excluded from the study. Four of the 24 had fetal structural abnormalities detected during their ultrasounds, 2 cystic hygromas, 1 congenital heart defect, 1 severe ventriculomegaly. Six cases had fetal chromosomal abnormalities other than Down syndrome, 3 Trisomy 18, 1 each of 45, X, 47,XXX and 47,XXY, and 14 were lost to follow up, leaving 351 women included in the final analysis. The characteristics of the study population are presented in Table 1. The median age of the participants was 35 years (range 15–45 years) and 59.1% were ≥ 35 years. All participants were of Thai ethnicity. A majority of the participants were gravidas of advanced maternal age who had amniocentesis (61.5%). Karyotyping was performed in women who underwent amniocentesis and newborn outcomes were determined after delivery. The median gestational age at birth of the euploid fetuses was 38 weeks 6 days with a median birth weight of 3,012 g. Ten of the 11 Down syndrome pregnancies were terminated before 24 weeks gestation.

**Table 1:** Characteristics of the study population

Parameter	Euploid fetuses (*n* = 340)	Fetuses with Down syndrome (*n* = 11)	*P* value
Maternal age (median, years)	35 (32,37.25)	39 (34.5,39.5)	0.0788
Gestational age at ultrasound (median, weeks)	17 (16,20)	18 (18,20)	0.0887
PT (mm) median (min,max)	2.73 (2.33,3.3)	4.8 (4.1,5.2)	< 0.001
NBL (mm) median (min,max)	5.4 (5,6.1)	1.4 (1.07,1.8)	< 0.001
PT/NBL ratio median (min,max)	0.5 (0.46,0.55)	3 (2.65,4.7)	< 0.001
NBL/PT ratio median (min,max)	2 (1.81,2.18)	0.33 (0.2,0.38)	< 0.001

*Min* Minimum, *Max* Maximum, *PT* Prenasal thickness, *NBL* Nasal bone length

All facial ultrasound markers of the fetuses with Down syndrome were statistically significantly different from the euploid fetuses as shown in Table 1. All fetuses with Down syndrome had abnormal PT/NBL and NBL/PT ratios. Hypoplastic NBL was found in 10/11 cases, while only 1 case had an absent nasal bone, in a mother who was 17 years old. Five of the 11 Down mothers had amniocentesis because of maternal age and only 1 of these 5 fetuses had a soft ultrasound marker, which was bilateral mild pyelectasis. Three cases underwent amniocentesis due to being found to be at high risk for a Down syndrome fetus from a quad test. Two cases underwent amniocentesis because of hypoplastic nasal bones and other soft ultrasound markers found during routine fetal anatomical scans. The last case had a fetus with mild pyelectasis and mild ventriculomegaly.

**Fig. 2:**
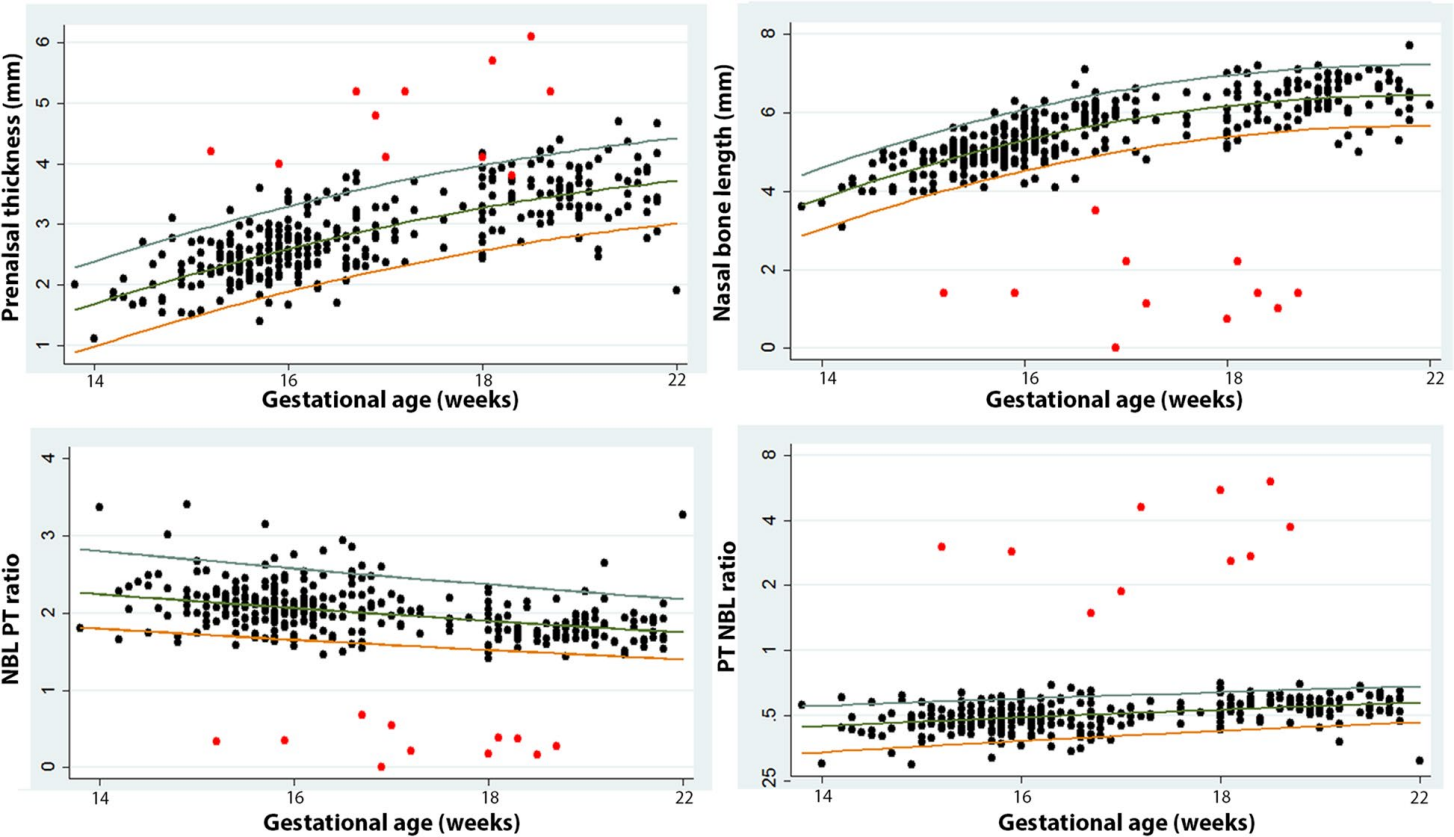
The distribution of facial ultrasound markers in the study euploid fetuses and fetuses with Down syndrome. **Legend:** PT, NBL, and PT/NBL and NBL/PT ratios according to gestational age of 340 euploid fetuses (black circles) and 11 fetuses with Down syndrome (red circles). *PT* prenasal thickness; *NBL* nasal bone length.

The mean PT, NBL, and PT/NBL and NBL/PT ratios of the euploid fetuses weekly from 14 to 22 weeks gestation are shown in Table 2. The mean PTs, NBLs, and PT/NBL ratios of our participants gradually increased while the NBL/PT ratios gradually decreased with advancing gestational age. Regression analysis was performed to predict the 5^th^ and 95^th^ percentiles of each facial parameter as shown in Table 3. All fetuses with Down syndrome had PTs, NBLs and NBL/PT ratios less than the 5^th^ percentile of euploid fetuses at the same gestational ages, while the PT/NBL ratio was higher than the 95^th^ percentile in all fetuses with Down syndrome (Fig. 2).

**Table 2:** The mean facial ultrasound markers of the euploid fetuses in our study during the second trimester

GA (Weeks)	PT (mm)	NBL (mm)	PT/NBL	NBL/PT
14	1.83	3.86	0.48	2.27
15	2.17	4.57	0.47	2.21
16	2.47	4.97	0.5	2.11
17	2.71	5.36	0.51	2.06
18	2.88	5.91	0.49	2.14
19	3.22	6.06	0.53	1.93
20	3.49	6.15	0.57	1.82
21	3.6	6.38	0.56	1.83
22	3.75	6.5	0.58	1.78

*GA* Gestational age (weeks), *PT* Prenasal thickness, *NBL* Nasal bone length

**Table 3:** The predictive properties of the 5^th^ and 95^th^ percentiles of each facial ultrasound marker

GA (weeks)	PT (mm)		NBL (mm)		PT/NBL Ratio		NBL/PT Ratio	
	**5**^**th**^	**95**^**th**^	**5**^**th**^	**95**^**th**^	**5**^**th**^	**95**^**th**^	**5**^**th**^	**95**^**th**^
14	1.06	2.47	3.18	4.74	0.34	0.56	1.80	2.80
15	1.40	2.81	3.75	5.31	0.36	0.57	1.74	2.71
16	1.71	3.11	4.25	5.81	0.37	0.59	1.69	2.63
17	1.99	3.40	4.67	6.23	0.39	0.60	1.66	2.55
18	2.24	3.65	5.02	6.58	0.40	0.62	1.59	2.47
19	2.47	3.88	5.29	6.85	0.42	0.63	1.54	2.40
20	2.66	4.07	5.48	7.04	0.43	0.65	1.50	2.33
21	2.83	4.24	5.61	7.16	0.45	0.66	1.46	2.27
22	2.97	4.38	5.65	7.21	0.46	0.68	1.42	2.21

*GA* Gestational age (weeks), *PT* Prenasal thickness, *NBL* Nasal bone length

The screening performance of the facial ultrasound markers is shown in Table 4. When using the predicted cut offs of NBL and NBL/PT ratio lower than the 5^th^ and PT and PT/NBL ratio higher than the 95^th^ percentiles of each gestational age, the NBL and PT/NBL and NBL/PT ratios showed sensitivity of 100% and specificity of 92%. When using a PT cut-Foff higher than the 95^th^ percentile, the sensitivity was lower than the other parameters at 91% but had a slightly higher specificity of 93%. When comparing the appropriate levels based on our study for percentiles (5^th^ and 95^th^) for each marker as estimated from the predicted mean markers that gave 100% sensitivity, the PT/NBL ratio had the lowest false positive rate of 6.5%, which gave an area under the curve (AUC) of 0.97. We had 0% false negatives when using the NBL cut off or NBL/PT ratio lower than the 5^th^ percentile or PT/NBL ratio higher than the 95^th^ percentile.

**Table 4:** Screening performance of the studied facial ultrasound markers for the fetuses with Down syndrome

	**PT**	**NBL**	**PT/NBL ratio**	**NBL/ PT ratio**
Sensitivity (%)	91	100	100	100
Specificity (%)	93	92	92	92
False positive (%)	6.76	7.6	6.5	7.6
False negative (%)	9.0	0	0	0
Positive predictive value	30	30	30	30
Negative predictive value	100	100	100	100
Positive likelihood ratio	13.44	13.08	13.08	13.08
Negative likelihood ratio	0.14	0.00	0.00	0.00
AUC	0.921	0.962	0.968	0.969

*AUC* Area under the curve, *PT* Prenasal thickness, *NBL* Nasal bone length

The Bland–Altman method was used to assess the intra- and inter-observer variability in measurements of the facial ultrasound markers of the operators in the study as shown in Table 5. Both the intra- and inter-observer variations of PT and NBL had high intraclass correlation coefficients (ICC) in both operators, with ICCs of 0.98 and 0.99 and inter-observer ICCs of 0.99 for both operators. The mean differences of PT and NBL were 0.01 mm (SD = 0.08) and -0.01 mm (SD = 0.11), respectively.

**Table 5:** Intraobserver and interobserver variability for the PT and NBL measurements

**Intraobserver variability (*n* = 100)**								
**Parameter**	**Operator 1**				**Operator 2**			
	**Mean difference ﻿(SD)**	**95% LoA**		**ICC**	**Mean difference (SD)**	**95% LoA**		**ICC**
PT	-0.04 (0.11)	-0.06 to -0.01		0.98	-0.02 (0.12)	-0.04 to 0.003		0.99
NBL	0.05 (0.16)	0.02 to 0.08		0.98	-0.03 (0.15)	-0.06 to -0.001		0.98
**Interobserver variability (*n* = 100)**								
	**Mean difference (SD)**		**95% LoA**				**ICC**	
PT	0.01 (0.08)		-0.003 to 0.03				0.99	
NBL	-0.01 (0.11)		-.022 to 0.02				0.99	

*SD* Standard deviation, *LoA* Limits of agreement, *ICC* intraclass correlation, *PT* Prenasal thickness, *NBL* Nasal bone length

## Discussion

Our study found as in other studies that a facial profile view of a fetus using a 2D ultrasound scan during the second trimester was not difficult to perform and could be done with a high level of reproducibility [11, 16] and high inter- and intra-observer agreements. A previous study reported that measuring facial ultrasound markers with 2D ultrasound as a screening protocol in the clinic had a comparable efficacy with 3D ultrasound [25]. However the use of 3D ultrasound can be limited in a difficult circumstance such as a low level of amniotic fluid [27], and if acquisition of a midsagittal facial plane image is not accomplished, the NBL measurement may be overestimated [28].

Our study found as in other studies that both PT and NBL in euploid fetuses increased with gestational age in the second trimester [11, 12, 15, 25, 29]. However, the predicted means and 5^th^ and 95^th^ percentiles of NB and PT in our euploid fetuses were found to be different during the second trimester among studies in Caucasian and Chinese participants [7, 11, 15, 30].

The mean PT in our study was slightly shorter than in other studies conducted in Caucasian and Chinese women [11, 12, 15, 24]. The mean NBL in the second trimester fetuses of our population was similar to the previously mentioned Chinese study [15], but slightly shorter than in euploid Caucasian fetuses [7, 12, 29]. The PT/NBL ratio of our euploid fetuses gradually increased during the second trimester, in contrast with other Caucasian and Chinese studies which showed a constant ratio during the second trimester [7, 15, 25, 30], while some of the Caucasian studies showed a gradual decrease during the second to early third trimesters [11, 12, 29]. This result of our study could be explained from the minimally shorter NBL of our euploid fetuses compared with Caucasian and Chinese fetuses at the same gestational age. The NBL/PT ratio in the euploid fetuses of our study gradually decreased during the second trimester, while one of the studies in Caucasians reported a gradual increase in this ratio during the second trimester [11]. The results of our study could be explained from the increase of PT in advancing gestational age while the NBL only minimally increased with advancing gestational age of our euploid fetuses.

Our study confirms that PT is significantly thicker and NBL significantly shorter in fetuses with Down syndrome in the second trimester. The PT/NBL ratio was higher and the NBL/PT ratio was lower in fetuses with Down syndrome in our study, similar to the findings from other studies in both Caucasian and Chinese fetuses [12, 15, 24, 25]. For the cut off of the PT/NBL ratio for Down syndrome screening, most previous studies used a cut-off at more than the 95^th^ percentile of the gestation, as in our study [7, 15]. However, some studies have suggested using a stable cut-off at the 95^th^ percentile of PT/NBL ratios because they found a stable PT/NBL ratio throughout the gestational periods [24, 25]. Ours is the first study in an Asian population to examine the NBL/PT ratio, and we found different values from a Caucasian study [11]. Our study found a small but significant decrease in the NBL/PT ratio in advancing gestation. Thus, we support using an NBL/PT ratio less than the 5th percentile of the gestation as a cut off for Down syndrome screening in our population. Our findings also indicate that the cut off values for facial ultrasound markers used for Down syndrome screening in the second trimester need to be constructed based on ethnicity.

Our study found that Down syndrome screening using these facial ultrasound markers in Asian fetuses was highly effective, as also found in previous studies in Caucasians, a high detection rate using the PT/NBL ratio was also found in a study of De Jong-Pleij et al. done in a high-risk pregnancy group [25]. However, we found a slightly higher false positive rate of 6.5% compared to De Jong-Pleij’s 5%. The previously mentioned study in Chinese patients found a low detection rate of the PT/NBL ratio of 46% and a 5% false positive rate, but when using the stable cut-off of 0.8 as in Caucasians the detection rate was 80.9% although with a substantially increased false positive rate of 21.6% [15]. These findings support the hypothesis that screening programs for Asian fetuses cannot use the Caucasian cut offs for facial ultrasound markers. Our study is the first to report on the performance of the NBL/PT ratio in an Asian population for Down screening and we found a high detection rate of 100% with a false positive rate of 7.6% and false negative rate of 0%, compared with the study of Szabo in Caucasians using this ratio which found a detection rate of 96.9% but with a lower false positive rate of 1.7% and false negative rate of 30.3% [11]. In assessing the screening performance of our study, the NBL and PT/NBL and NBL/PT ratios had 0% negative predictive value with AUCs of 0.96, 0.97 and 0.97, respectively. We conclude that the use of facial ultrasound markers should be encouraged to increase the detection rate of Down syndrome screening in the second trimester in our population.

With current advances in prenatal screening for Down syndrome, cell-free DNA has become the most effective Down screening method [31]. However, this screening method is costly, and limited to only some centers in low-resource or developing countries [32]. There is evidence showing that using multiple soft ultrasound markers can increase the detection rate of Down syndrome [9]. However, using multiple soft ultrasound markers for Down syndrome screening is not universal because of a lack of expertise in many centers and the longer time required. A related study found that adding genetic sonogram screening in pregnancies identified as low risk for a Down syndrome fetus by cell-free DNA had a low positive predictive value [33]. To optimize the screening performance for Down syndrome, particularly in some developing countries in which maternal serum biomarkers are currently used but have lower detection rates than expected, such as in our setting [34], our study supports the idea of adding facial ultrasound markers in groups at moderate risk for Down syndrome to maternal serum biomarkers. As a previous study using only absent or hypoplastic nasal bone for Down syndrome screening reported a low detection rate for Down syndrome in Asian fetuses [35], adding multiple facial ultrasound markers should increase the detection rate of Down syndrome screening in our populations [36]. As another potential benefit of using facial ultrasound markers screening, a recent study found that the presence of isolated facial ultrasound markers such as a hypoplastic nasal bone could indicate other fetal chromosomal abnormalities and pathogenic copy number variants [37]. In such cases, if abnormal facial ultrasound markers are found, then an increased risk of Down syndrome would indicate the patient should be considered for amniocentesis.

Our study was done in a homogeneous group of Thai women. This is the first study done in an Asian group to assess the appropriate reference ranges of potential facial ultrasound markers, and we found high performance in screening for Down syndrome in the second trimester. Our suggested reference ranges can be combined with the other screening methods that are offered in the first or second trimester as an adjunct test to increase the detection rate of Down syndrome screening or at places that lack fetal medicine specialists to perform genetic sonograms as a universal screening program. From the variety of measurement techniques available, in our study we chose 2D ultrasound, which is widely used, practical for general obstetricians, and takes less time to learn compared with the 3D technique. Also, these facial parameters had high inter-observer agreement for the measurements. All of these facts support our suggestion that fetal facial ultrasound markers should be promoted as a routine or adjunct to a screening program by general obstetricians in our population.

Our study had some limitations. First, the number of fetuses with Down syndrome was smaller than in other studies. Also, our study was done only in a high-risk population, thus the results may not reflect the actual proportions in a mixed-risk population. We also had a limited number of cases in some gestational age periods, and thus our findings may not reflect the normal distribution of facial parameters in euploid fetuses.

## Conclusion

To measure fetal facial ultrasound markers with 2D ultrasound in the second trimester is not difficult, has highly consistent results among operators and had a high performance for Down syndrome screening in our population. However, the reference ranges of facial ultrasound markers need to be constructed based on ethnicities.

## Data Availability

The datasets analyzed during the study are available from the corresponding author upon reasonable request.

## Abbreviations

PT: Prenasal thickness
NBL: Nasal bone length
PT/NBL ratio: Prenasal thickness to nasal bone length ratio; nasal bone length to prenasal thickness ratio
2D ultrasound: 2-Dimensional ultrasound
ICC: Intraclass correlation coefficient
min: Minimum
max: Maximum
